# *In vitro* myelination using explant culture of dorsal root ganglia: An efficient tool for analyzing peripheral nerve differentiation and disease modeling

**DOI:** 10.1371/journal.pone.0285897

**Published:** 2023-05-24

**Authors:** Yurika Numata-Uematasu, Shuji Wakatsuki, Yuka Kobayashi-Ujiie, Kazuhisa Sakai, Noritaka Ichinohe, Toshiyuki Araki

**Affiliations:** 1 Department of Peripheral Nervous System Research, National Institute of Neuroscience, National Center of Neurology and Psychiatry, Tokyo, Japan; 2 Department of Ultrastructural Research, National Institute of Neuroscience, National Center of Neurology and Psychiatry, Tokyo, Japan; Aix Marseille University, FRANCE

## Abstract

Peripheral nerves conducting motor and somatosensory signals in vertebrate consist of myelinated and unmyelinated axons. *In vitro* myelination culture, generated by co-culturing Schwann cells (SCs) and dorsal root ganglion (DRG) neurons, is an indispensable tool for modeling physiological and pathological conditions of the peripheral nervous system (PNS). This technique allows researchers to overexpress or downregulate molecules investigated in neurons or SCs to evaluate the effect of such molecules on myelination. *In vitro* myelination experiments are usually time-consuming and labor-intensive to perform. Here we report an optimized protocol for *in vitro* myelination using DRG explant culture. We found that our *in vitro* myelination using DRG explant (IVMDE) culture not only achieves myelination with higher efficiency than conventional *in vitro* myelination methods, but also can be used to observe Remak bundle and non-myelinating SCs, which were unrecognizable in conventional methods. Because of these characteristics, IVMDE may be useful in modeling PNS diseases, including Charcot Marie Tooth disease (CMT), *in vitro*. These results suggest that IVMDE may achieve a condition more similar to peripheral nerve myelination observed during physiological development.

## Introduction

Peripheral nerves conducting motor and somatosensory signals in vertebrate consist of myelinated and unmyelinated axons [1]. Myelin, a lipid-rich ionic insulator that wraps around axons, enables rapid saltatory conduction of neuronal electrical signals along the axon and supports axonal metabolism [2]. In the peripheral nervous system (PNS), myelination is accomplished by Schwann cells (SCs), a type of glial cells specifically located in PNS [3]. Disruption of myelination in the PNS results in demyelinating diseases, such as hereditary motor and sensory neuropathies [4]. Also in the PNS, SCs are present as non-myelinating cells. Small diameter sensory axons are unmyelinated and surrounded by a specialized type of non-myelinating SCs, termed Remak SCs [3].

To evaluate physiological/pathological conditions of neurons and SCs of the PNS, it is essential to observe them as myelinated nerves even in culture, because SCs in dissociated culture do not show a fully differentiated phenotype [5–7]. Therefore, co-cultures of peripheral sensory neurons from dorsal root ganglia (DRG) and SCs from peripheral nerves for myelination in culture (*in vitro* myelination) have often been used for modeling physiological/pathological conditions of the PNS [8–11]. *In vitro* myelination is usually established by applying cultured rat SCs generated from early postnatal sciatic nerves onto established neuron culture generated from E15-16 rat DRG neurons, followed by initiation of myelination by adding ascorbic acid to the culture media [8–10, 12]. A possible alternative method is to generate neuron-SC co-culture using mouse/rat embryo DRG explants, in which SCs in the DRGs migrate out of DRGs, proliferate along the axons, and myelinate in response to added ascorbic acid [13, 14]. While this method may be simpler than the former one, because neurons and SCs do not need to be separately prepared prior to the initiation of the co-culture, DRGs require treatment by a proteolytic enzyme for dissociation, and the resultant culture is similar to the former one with apparently similar myelination efficiencies.

Here we identified that *in vitro* myelination using DRG explant (IVMDE) has additional benefits as a culture model of peripheral nerve myelination. We found that the frequency of myelination is consistently greater in *in vitro* myelination culture using DRG explants, rather than *in vitro* myelination using dissociated neurons/SCs. In addition, EM analysis revealed the formation of Remak bundle-like structures by non-myelinating SCs in IVMDE. We found by using IVMDE that axon-SC interaction was achieved by the formation of compact myelin, which may be necessary for axonal maturation characterized by the increase of axonal diameter. These characteristics of IVMDE suggest that the IVMDE may achieve a condition closer to peripheral nerve myelination during physiological development, and may be more suitable for modeling PNS disorders in culture.

## Materials and methods

### Ethics statement

All the animal experiments were performed in accordance with the guideline of National Center of Neurology and Psychiatry (NCNP) and under written approval by the Animal Care Committee of NCNP (Approval No. 2020027).

### Mice

For genotyping of trembler-NCNP mice [15], transgenic progeny were identified with polymerase chain reaction (PCR) on genomic DNA extracted from a tail tissue fragment using the following primer set; forward 1: TCAGGGACAGTACCAGAGCTCA, forward 2: CCGTATTTCTCGATCACACAC and reverse: GAGCTAGTTAGCTGCTGGACA. PCR was conducted by 32 cycles of 94°C for 1 min, 60°C for 1min and 72°C for 1min.

### DRG explant culture

DRGs were excised from E14 Sprague-Dawley (SD) rat or E12 mouse embryos. The entire ganglia were plated on 24 well plates coated with poly-L lysine (PLL) (Sigma Aldrich) and cultrex mouse laminin I (R&D systems), and cultured with MACS ^R^ Neuro Medium (Miltenyi Biotec) supplemented with 10% fetal bovine serum (FBS) (Sigma Aldrich), 1:200 Glutamax (Thermo Fischer Scientific), 50 U/ml Penicillin/Streptomycin (Thermo Fischer Scientific) and 100ng/ml 2.5S nerve growth factor (NGF) (COSMO BIO). Sixteen hours later, the medium was changed to MACS ^R^ Neuro Medium supplemented with 1% MACS^R^ NeuroBrew^R^-B21 (Miltenyi Biotec), 1:200 Glutamax, 50 U/ml Penicillin/Streptomycin and 100 ng/ml 2.5S NGF. At day 5, the medium was replaced DMEM supplemented with 10% FBS, 1:200 Glutamax, 50 U/ml Penicillin/Streptomycin and 100 ng/ml 2.5S NGF, and thereafter half of the medium was changed every other day. On day14, 50 μg/ml of L-ascorbic acid (Sigma Aldrich) was added to the medium to initiate myelination. For EM analysis, DRGs were cultured on Cell Disk LF (Sumitomo Bakelite Co.) coated PLL and cultrex mouse laminin I.

### Primary Schwann cell culture

SCs were prepared from sciatic nerves of postnatal day 2 SD rats. Contaminating fibroblasts were removed from culture by treating the culture with 10 μM cytosine arabinoside (Wako) for 48 h and by complement-mediated cytolysis using anti-Thy1.1 (Serotec, Oxford, UK) and rabbit complement (Cappel laboratories, Cochranville, PA, USA). SCs were propagated on PLL-coated plates in DMEM supplemented with 10% FBS, 2 μM forskolin (Sigma Aldrich), and 20 ng/ml glial growth factor (Pepro Tech). For myelination assay using the co-culture with dissociated DRGs, primary SCs were used after 3 or more passages.

### *In vitro* myelination in dissociated culture

Dissociated neuron cultures were prepared from DRGs obtained from E15 SD rat pups and maintained in 24-well plate coated PLL and laminin (2 x 10^5^ cells per each well) in Neurobasal medium supplemented with B21, Glutamax, 50 U/ml Penicillin/Streptomycin and 100 ng/ml 2.5S NGF. Non-neuronal cells were removed by treating the cultures with media containing 5-fluorodeoxyuridine (Sigma Aldrich) for 10 days. SCs (3 x 10^5^ cells per well) were then plated onto the established neuronal culture. Ten days later, 50 μg/ml ascorbic acid was added to the mixed cultures of SCs and neuron for the initiation of myelination.

### Immunocytochemistry

The cells were fixed using 4% paraformaldehyde (Wako) in PBS for 10 min, permeabilized with 0.5% Triton X-100 (Wako) for 30 min, and treated with 3% bovine serum albumin to block non-specific binding. Cells were further incubated with the primary antibodies for 16 h at 4°C followed by visualization using the appropriate secondary antibodies labeled with Alexa-488 and -594 with 4’, 6-diamidino-2-phenylindole (DAPI). The primary antibodies used include: mouse anti-myelin basic protein (MBP) (SMI-99, COVANCE); anti-βⅢ Tubulin (COVANCE); mouse anti-myelin associated glycoprotein (MAG), clone513 (Millipore); rabbit anti-neurofilament M (Merk). Alexa Flour 488-conjugated anti-rabbit IgG (Thermo Fisher Scientific) and Alexa Flour 594-conjugated anti-mouse IgG (Thermo Fisher Scientific) were used as secondary antibodies for immunocytochemistry. For quantification, myelination profiles visualized with MBP staining was quantified in 5 randomly selected fields using a 20x objective lens and the number of myelinated nerve fibers per arbitrary unit area was calculated.

### Electron microscopy (EM)

Cells were fixed with 2% paraformaldehyde and 2% glutaraldehyde (Nisshin-EM) in 0.1 M PB for O/N at 4°C. The samples were treated with 1% osmium tetroxide in 0.1M cacodylate buffer for 30 min. The samples were stained with 3% uranyl acetate for 60min, serially dehydrated, and embedded in epon812 (TAAB). For ultrastructural analysis, 70nm ultrathin sections were made and observed under a transmission EM (Tecnai Sprit, FEI/Thermo Fisher Scientific).

### Quantification of axonal number and diameter

Quantification of the axonal number and diameter was performed by counting/measuring the numbers and diameters of axons in 10 randomly selected areas (one area = 332.5 μm^2^) of the DRG explants.

### Quantitative RT-PCR

Total RNA was extracted from the cells using the RNeasy MiniKit (Qiagen) and cDNA was made using ReverTra Ace reverse transcriptase (TOYOBO). Transcript levels were analyzed on the Applied Biosystems Prism model 7300 using a standard SYBR green detection protocol. The sequences of the PCR primers using the SYBR green method are as follows: MBP forward, 5’-ACTCACACACAAGAACTACCCA-3’, MBP reverse, 5’-AGCTAAATCTGCTGAGGGACA-3’, GAPDH forward, 5’-CCACGGCAAGTTCAACGGCACAGT-3’, and GAPDH reverse, 5’-CAGCGGAAGGGGCGGAGATGAT-3’. According to SYBR green RT-PCR methods, the fluorescence data were quantitatively analyzed using serial dilutions of control samples included in each reaction to produce a standard curve.

### Statistics

All data were graphically presented as mean ± SEM. For comparisons of efficacy in dissociated culture model and IVMDE culture model, statistical comparisons were made using exact Wilcoxon rank sum test. For analysis of unmyelinated and myelinated axon calibers in IVMDE from wild type rat and mice, statistical comparisons were done using two-sided Kolmogorov-Smirnov test. For analysis of the number and length of myelin profiles using DRGs from wild type and Trembler-NCNP mice, statistical comparisons were performed using Student’s t-test. *p*<0.05 is considered statistically significant.

## Results

### Characterization of IVMDE as an efficient tool for modeling PNS development

Critical events during the initial stages of DRG explant culture include attachment of the explants to the culture dish surface, neurite extension, and SC proliferation. Previous reports described “dissociated explants” by treating DRGs with trypsin, presumably for proper tissue attachment and destruction of fibroblast capsule surrounding the DRG for better neurite extension and SC migration [13, 14]. Svenningsen et al. described use of enriched culture surface matrices (Matrigel and PLL) and culture media without serum for sufficient neurite extension and SC migration without extensive growth of fibroblasts [14]. We found that, by dissecting out DRGs from E12 mouse /E14 rat embryos, DRG explants attach on the culture dish in the culture media and extend neurites radially with migrating SCs, just by placing DRGs without enzymatic digestion on culture dish surface coated with PLL and laminin I. We also found that fibroblasts do not overgrow in our IVMDE culture by using serum-containing culture media, presumably because SCs, which migrate out of DRGs with extending neurites, easily outgrow fibroblasts at this developmental stage.

**Fig 1:**
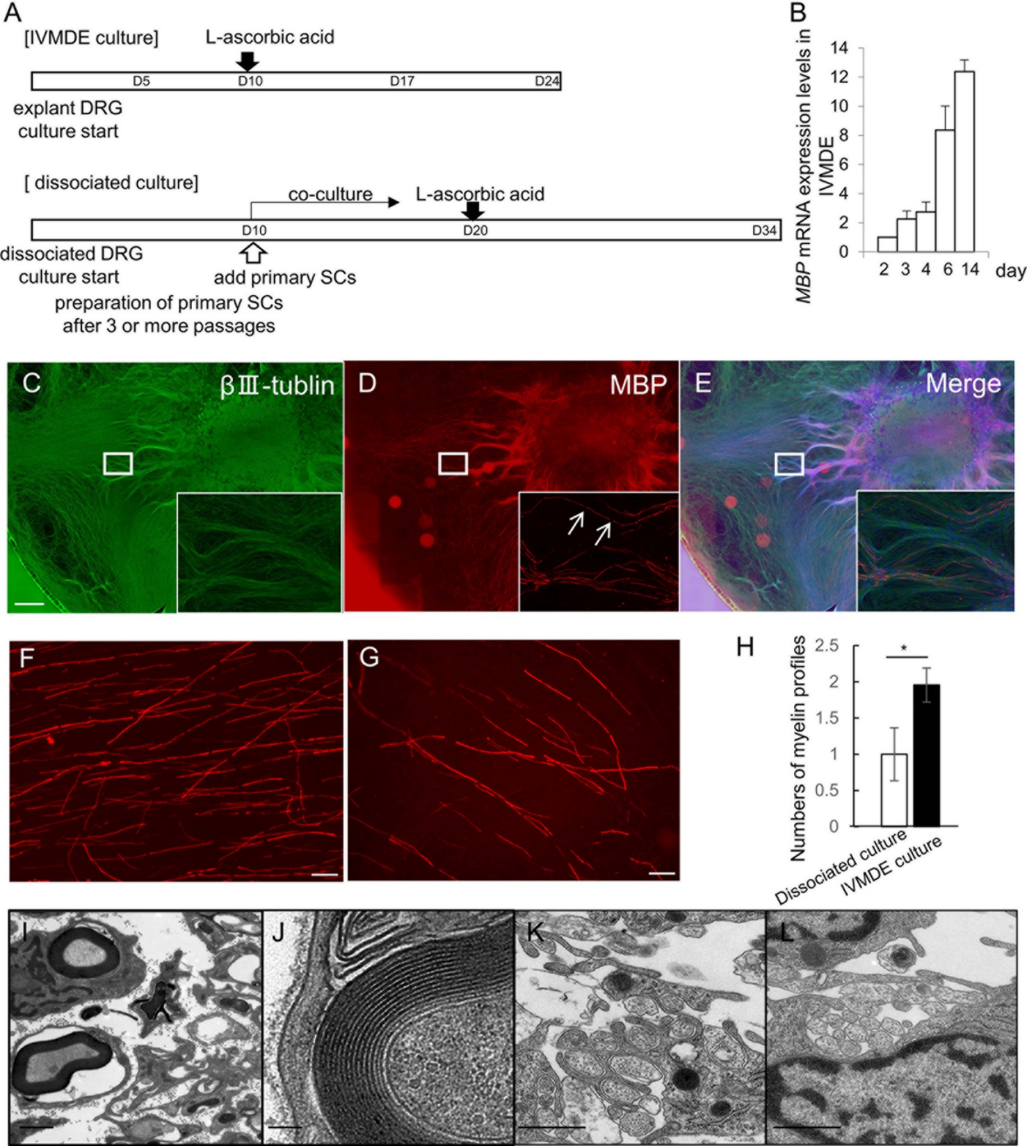
Schematic representation of culture schedule and characterization of IVMDE. (A). Schematic representation of culture schedules for rat/mouse IVMDE in comparison to the typical *in vitro* myelination protocol using rat dissociated neurons. (B) MBP mRNA expression levels in rat IVMDE after initiation of myelination by ascorbic acid. MBP expression levels at indicated days after initiation of myelination are shown normalized to GAPDH expression relative to the expression on day 2. The data from each experiment are shown as S1 File. (C-E) Representative immunocytochemistry photomicrographs of rat IVMDE at 14 days after induction of myelination by ascorbic acid to detect βⅢ-tubulin depicted in green, and MBP depicted in red are shown. Scale bar = 1mm. Insets show high-power view of the rectangular area in each panel. Arrows in (D) indicate nodes of Ranvier identifiable in light microscopy. (F, G) Representative immunocytochemistry photomicrographs of MBP in IVMDE (F) and conventional dissociated culture (G), both using rat cells. Scale bar = 50μm. (H) The number of myelin profiles in *in vitro* myelination culture using dissociated neurons and in IVMDE are shown relative to the number in dissociated neuron experiments. The myelination profiles were counted in 5 microscopic fields randomly selected from 5 DRG cultures (1 field per each DRG culture) in 3 independent culture experiments. *p<0.01. The data from each experiment are shown as S1 File. (I~L) Representative photomicrographs for EM analysis of rat IVMDE culture showing myelinated (I, J) and non-myelinated (K, L) axons/SCs are shown. Scale bar = 1μm (I, K, L), 100 nm (J).

Our protocol for IVMDE culture is summarized in Fig 1A. As the timeline of this chart shows, it usually takes around 10 days from tissue harvest to the initiation of myelination by addition of ascorbic acid to culture medium for IVMDE culture. The time course of the myelination after initiation with ascorbic acid supplementation is similar to that seen in dissociated cultures, and gradual increase of MBP expression was observed after the initiation of myelination (Fig 1B). We observed that the myelination tends to start from the region close to the neuronal cell bodies, and extends to the periphery (Fig 1C–1E). Some of the myelin profiles aligned along the axons separated by narrow gaps which are compatible with the light microscopic appearance of nodes of Ranvier (Fig 1D). We realized that the frequency of myelination seems higher by using DRG explants than by using dissociated cells (Fig 1F and 1G). To compare the myelination frequency quantitatively, we counted the number of myelination profiles visualized by MBP immunoreactivity in IVMDE versus *in vitro* myelination using dissociated neurons at 14 d after initiation of myelination. As shown in Fig 1H, we found that myelination is more frequently observed using DRG explant than by dissociated neurons culture.

To further characterize IVMDE, we performed detailed morphological examination of the culture using EM. We confirmed that MBP-positive structures visualized by immunocytochemistry corresponded to tightly wrapped myelination (Fig 1I and 1J). In addition to the myelinated axons, we also found Remak SC-like structures, in which several unmyelinated axons were surrounded by non-myelinating SCs (Fig 1K and 1L). Such unmyelinated nerves are not usually noted in co-cultures of dissociated DRGs and primary SCs.　These observations suggest that parallel array of axons in DRG explant culture may allow radial sorting of SCs to differentiate them into Remak SCs, and that IVMDE might achieve a culture condition more similar to physiological condition during peripheral nerve development.

### Maturation of IVMDE is characterized by myelination progression and changes in axon size

**Fig 2:**
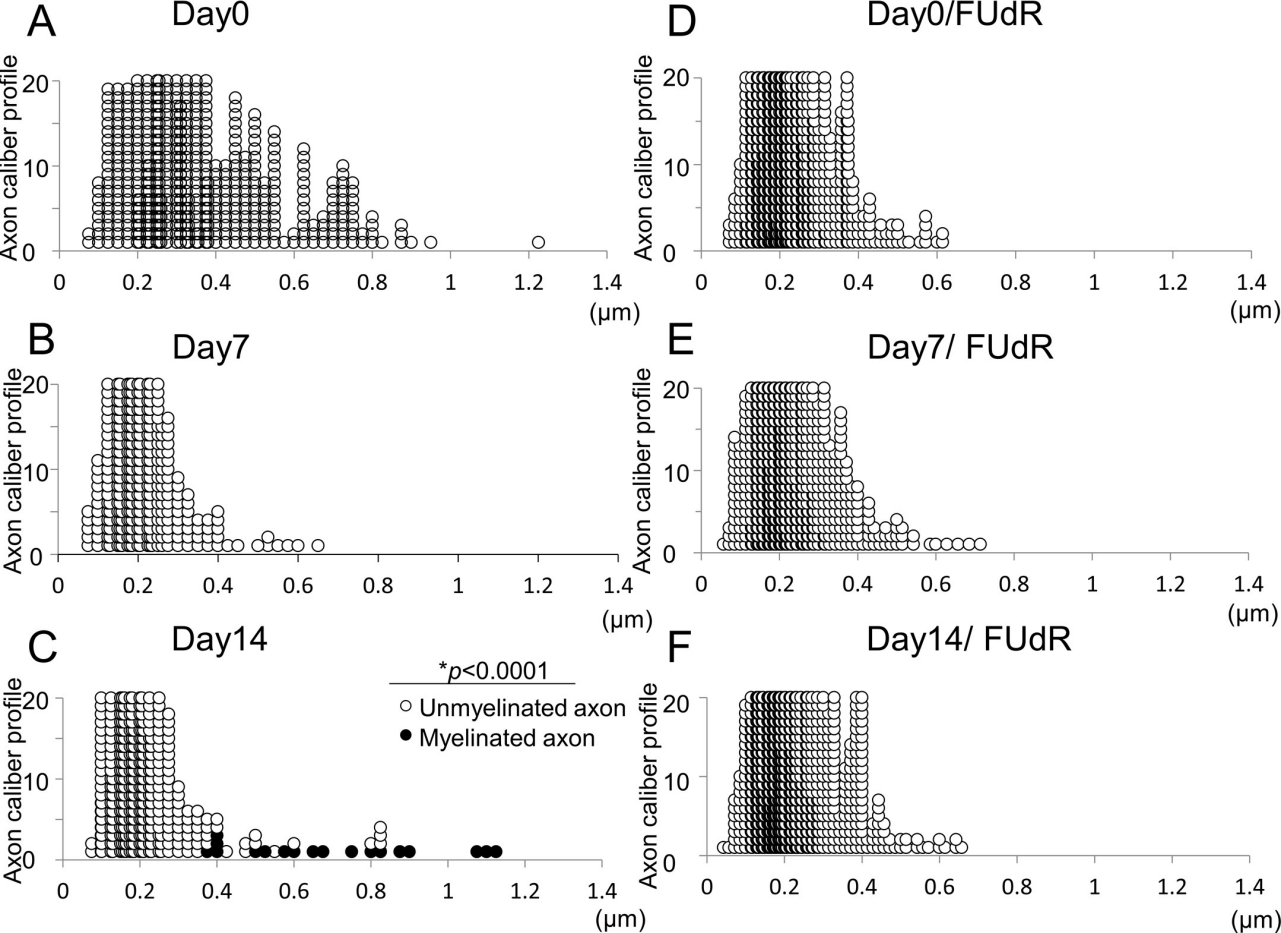
Axonal calibers change by maturation of IVMDE. Changes in axonal caliber in rat IVMDE after the initiation of myelination were analyzed by EM. Caliber of each axon identified in 10 randomly selected fields by EM at 0 (A, D), 7 (B, E) and 14 days (C, F) after the initiation of myelination without (A~C) or with (D~F) an antimitotic reagent (5-fluorodeoxyuridine) is plotted. Axonal calibers were measured in 10 microscopic fields randomly selected from 3 DRG cultures in 2 independent culture experiments for A~C, and in 10 microscopic fields randomly selected from 2 DRG culture experiments for D and from 1 DRG culture experiment for E and F. Black and white circles represent myelinated and non-myelinated axons, respectively. Note that all large diameter axons are myelinated.

Based on the hypothesis that IVMDE may be able to more closely mimic physiological conditions, we further examined how IVMDE culture develops and matures by utilizing EM. In the PNS, larger caliber axons are wrapped by myelin, whereas smaller axons remain unmyelinated or become thinly myelinated [1, 16]. To demonstrate the differentiation process of different sized axons in IVMDE cultures, we monitored the diameter of axons in IVMDE during maturation of the culture. We found that axon diameters vary in size without the initiation of myelination. After the initiation of myelination, the distribution of axon size remains diverged so that the caliber of unmyelinated axons remains small, while myelinated axons show larger calibers (Fig 2A–2C). We did not observe major axonal degeneration after induction of myelination (data not shown). It is possible that some axons may be degraded in the course of IVMDE culture, but that should not affect the axonal caliber profile. This variation in axon diameter was not observed when non-neuronal cells were removed with an antimitotic reagent, suggesting that culture components, including non-neuronal cells and SCs, must play a role in the size determination of axons (Fig 2D–2F). These observations collectively suggest that axons mature in conjunction with the progression of myelination in IVMDE.

### Application of IVMDE for modeling CMT in culture

**Fig 3:**
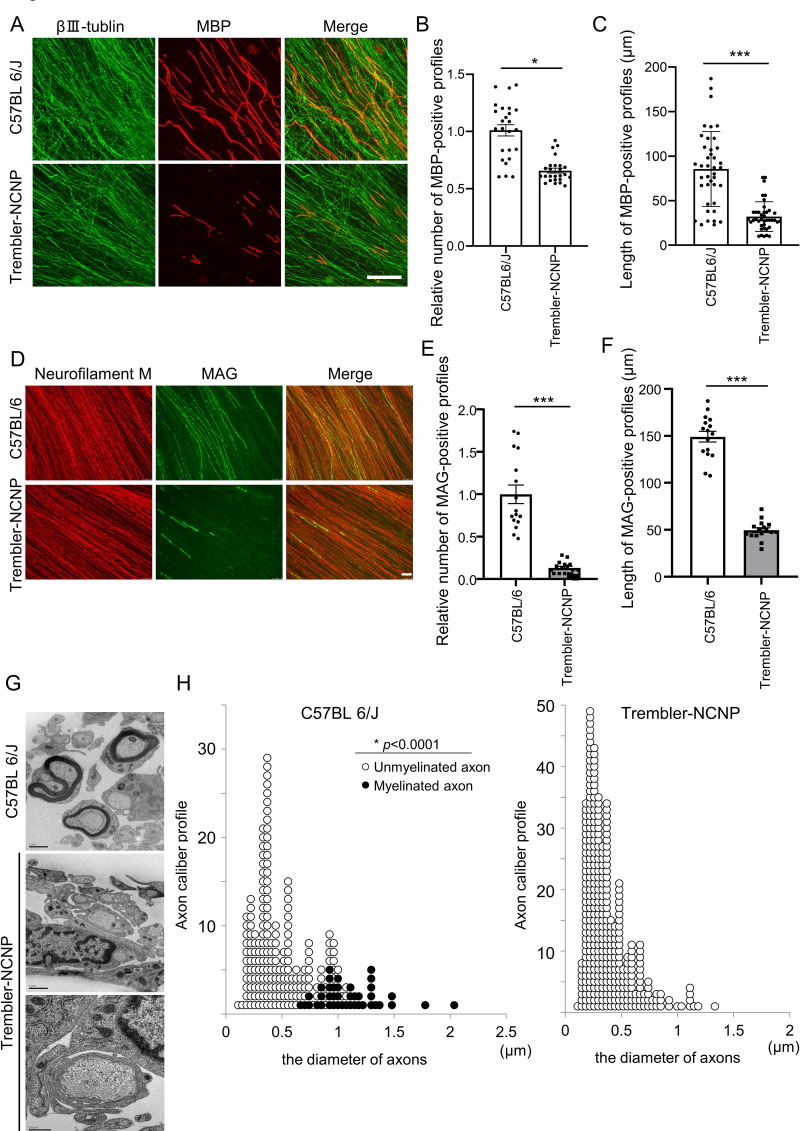
IVMDE using Trembler-NCNP mouse DRGs shows dysmyelination phenotype. (A) Representative immunocytochemistry photomicrographs showing βⅢ-Tubulin and MBP depicted respectively in green and red in wild type mouse and Trembler-NCNP mouse at 14 days after initiation of myelination in IVMDE. Scale bar = 100 μm. (B, C) Bar graph showing the number (B) and length (C) of myelin profiles visualized by MBP immunoreactivity in IVMDE using DRGs from wild type and Trembler-NCNP mice. The numbers in (B) are shown relative to the mean number from the experiments using wild type mice. Myelination profiles were counted in 25 randomly selected microscopic fields in 3 independent culture experiments performed for each genotype. Statistical analysis was performed by Student’s t-test. Asterisks indicate significant difference (*p<0.05, ***p<0.001). (D) Representative immunocytochemistry photomicrographs showing neurofilament and MAG depicted in red and green, respectively, in wild type mouse and Trembler-NCNP mouse at 14 days after initiation of myelination in IVMDE. Scale bar = 50 μm. (E, F) Bar graphs showing the number (E) and length (F) of myelin profiles, visualized by MAG immunoreactivity in IVMDE using DRGs from wild type and Trembler-NCNP mice. The numbers in (E) are shown relative to the mean number from the experiments using wild type mice. Myelination profiles were counted in 4 randomly selected microscopic fields in 4 independent culture experiments performed for each genotype. Statistical analysis was performed by Student’s t-test. Asterisks indicate significant difference (***p<0.001). (G) Representative photomicrographs for EM analysis of IVMDE using DRGs from wild type and Trembler-NCNP mice. Scale bar = 1 μm (upper and middle panel), 0.5 μm (lower panel). (H) Analysis of axon size in IVMDE using DRGs from wild type and Trembler-NCNP mice. The caliber of each axon identified in 10 randomly selected fields by EM at 14 days after the initiation of myelination is plotted. *p<0.0001.

As stated above, conventional *in vitro* myelination culture using dissociated DRG neurons has been performed almost exclusively in rats, because it is empirically known that the myelination efficiency is low using cells from species other than rats. In addition, the culture protocol for proliferation/maintenance of SCs is established for rat, but not for other species. The current protocol for IVMDE, on the other hand, enables myelination culture in mice as demonstrated in Fig 3. To apply the current protocol to disease modeling, we generated IVMDE culture from Trembler-NCNP mice, a CMT type 1 disease model. We found that myelin (visualized by MBP immunocytochemistry) is formed in IVMDE culture using Trembler-NCNP mice-derived DRG explants, but the number of myelination profiles is significantly smaller than that in wild type mice-derived culture (Fig 3A and 3B). In addition, the length of myelination profiles was significantly shorter and intensity of MBP immunoreactivity of myelination profiles was weaker in IVMDE culture using Trembler-NCNP mice-derived DRG explants than those of wild type controls (Fig 3C). These results suggest that the length of myelination profile and intensity of MBP immunoreactivity could be used as indicators reflecting myelination maturity. To further demonstrate the immaturity in myelination capacity of Trembler-NCNP mice-derived DRG explants, we also examined MAG immunoreactivity. We found that MAG-positive profiles in IVMDE culture using Trembler-NCNP mice-derived DRG explants appear as short fragments, while those in wild type mouse-derived cultures show uninterrupted linear patterns along radially extended axons (Fig 3D–3F). To further characterize myelination using Trembler-NCNP mouse DRGs in IVMDE culture, we performed morphological analysis using EM (Fig 3G). We found only loose wrapping of axons in IVMDE culture using Trembler-NCNP mouse DRGs, and not compact myelin as observed using wild-type mouse DRGs (Fig 3G, lower panel). Analysis of the axon caliber profile in IVMDE culture using Trembler-NCNP mouse DRGs revealed that the diameter of axons was distributed in the small-size range, while the axon caliber profile from the culture of wild type mouse DRGs was similar to that of wild type rat-derived DRG explants. IVMDE culture using wild type mouse DRGs also showed typical polarized distribution with the unmyelinated and myelinated axons having smaller and larger calibers, respectively (Fig 3H). These results suggest that myelination profiles observed with MBP immunostaining of IVMDE culture using Trembler-NCNP mouse DRGs may represent loose wrapping of axons, which is not sufficient for inducing maturation of axons.

## Discussion

Here we established an efficient protocol for peripheral myelination culture. IVMDE does not require separate preparation of neuron and SCs from different age animals; but allows SC precursors in DRGs to migrate out and associate with axons as they extend from the cell bodies, so it takes much less time and effort before initiating myelination by adding ascorbic acid to the culture medium. We also found that myelination is more frequent in IVMDE. IVMDE allows investigators to observe Remak SC-like structures and axonal morphological differentiation as well, suggesting that IVMDE might achieve a condition more similar to physiological development *in vivo* than that seen when dissociated neuron cultures are used.

One of the key factors that enabled us for successful establishment of the simple IVMDE protocol is selecting E12 (mouse) /E14 (rat) for harvesting DRGs for culture. Some previous reports for establishment of myelination culture using DRG explants describe proteolytic enzyme treatment of DRGs [13, 14], presumably for proper tissue attachment on the culture dish surface and destruction of fibroblast capsule surrounding DRGs. By collecting DRGs from younger embryos in which fibroblasts can be separated from each other easily without enzymatic digestion [17], we were able to establish a more simplified protocol. Other reports described addition of separately prepared SCs on top of the neuron-only culture for *in vitro* myelination experiments [18]. Such procedures require to add forskolin and/or pituitary extract to the co-culture to facilitate SC proliferation, presumably because added SCs do not proliferate well without additional mitogenic stimulations. This is in sharp contrast to our protocol, in which SCs, which migrate out of the DRG explant, proliferate along neuronal axons dependent on factors derived from axons without externally applied reagents, prior to the initiation of myelination. It has been reported that SCs at their precursor stage in E12 mice/E14 rats survive, proliferate, and differentiate depending on signals from axons [19]. By performing IVDME culture using DRGs dissected out of E12 mice/E14 rats, SCs probably proliferate at their maximal rates during the first several days in culture [20], and subsequently differentiate in a time course similar to the physiological development.

The value of *in vitro* myelination culture is multifaceted. It allows us to evaluate the effects of genetic modification or chemical compound intervention on SCs and/or neurons relatively easily as a cell culture experiment. Quantitative comparison of different experimental condition can be easily performed, usually by quantifying the number of myelination profiles. Here we found that myelination culture using Trembler-NCNP mouse-derived DRG explants exhibits shorter myelination profile length with weaker MBP immunoreactivity than control wild type mouse-derived culture. These observed differences presumably result from the loose folding of myelin wrapping around axons in Trembler-NCNP mouse nerves, and reflect CMT pathology [21]. Short myelination profiles with weak MBP intensity could easily be disregarded in *in vitro* myelination assays with dissociated neurons, because the myelination frequency is generally lower in that paradigm. This suggests that the high myelination efficiency observed in IVMDE could generate new indexes for evaluating myelination allowing discovery of therapeutic interventions that normalize the myelin profile length and MBP intensity as seen in many peripheral nerve diseases causing dysmyelination.

Liu et al. showed the myelination figures are observed by EM analysis [22], but we never observed good myelination figures in samples derived from Trembler-NCNP mice. We also noticed that intensity of MBP immunoreactivity of myelination segments in light microscopic level analysis of the Trembler mouse-derived myelination cultures in the previous report [22] look comparable to that observed in wild type mouse-derived samples, although the comparison is not quantitative. This is in sharp contrast to our observations, in which MBP immunoreactivity is much less intense in Trembler-NCNP mouse-derived samples. Based on this difference, we presume that the MBP positive figures in our Trembler-NCNP mouse-derived samples correspond to SCs associated with but not tightly wrapping axons. Liu et al. described that the MAG levels were comparable between the Trembler J and wild type explant cultures [22]. On the other hand, we found that MAG immunoreactivity in Trembler-NCNP-derived DRG explants shows quite different patterns compared with the culture from wild type control (Fig 3D), suggesting that MAG expression in IVMDE culture from Trembler-NCNP mice is lower than that from wild type. This difference in MAG staining may also reflect the difference that causes decreased myelination in Trembler-NCNP mouse-derived myelination culture compared with the myelination using Trembler J mouse-derived cells reported in Liu et al. [22] observed in EM analysis. We are not exactly sure where the differences between our observations and those by Liu et al. [22] are derived from. The difference in genetic backgrounds of Trembler mice (i.e., Trembler J, bearing point mutation in PMP22, vs. Trembler-NCNP, which is a deletion mutant lacking a 17kb region in pmp22 gene containing exon IV) may be responsible for the different degrees in myelination in culture.

Here we found that axonal morphological differentiation including changes in diameter may be an event requiring myelination. We observed large-sized axons are all myelinated after the induction of myelination, which is compatible with the previously reported observations, while there are some large-sized unmyelinated axons prior to the myelination induction [23]. Previous reports suggested that large-sized axons become myelinated in the radial sorting process during development [24]. Our current observations seem to suggest, in addition to the putative axonal caliber-dependent myelination mechanism, that axonal caliber determinants may be different before and after the myelination induction. We speculate that the axonal caliber increase prior to the induction of myelination is caused as a result of growth. At this stage, some axons grow faster than others, so axonal calibers can vary. On the other hand, the increase after the induction of myelination is probably a part of axonal differentiation. Wrapping by SCs is likely to be required for differentiation of large caliber axons. Determination of SC-axon signals critical for axonal differentiation and subsequent confirmation of their significance in more physiological contexts will be required.

## Supporting information

S1 File (ZIP)

## Abbreviations

PNS: peripheral nervous system
SC: Schwann cells
DRG: dorsal root ganglion
CMT: Charcot-Marie-Tooth
IVMDE: *in vitro* myelination using DRG explant
PCR: polymerase chain reaction
PLL: poly-L lysine
FBS: fetal bovine serum
NGF: nerve growth factor
MBP: myelin basic protein
DAPI: 6-diamidino-2-phenylindole
MAG: myelin associated glycoprotein
